# Physicochemical characteristics of *Pangasius* sp. skin-gelatin-based-edible film enriched with silver nanoparticles

**DOI:** 10.12688/f1000research.129024.1

**Published:** 2023-02-10

**Authors:** Rahmi Nurdiani, Asep A. Prihanto, Muhamad Firdaus, Faridatul Nur Aini, Fajrin Adin Nabilah, Rosnita A Talib, Nurul Huda

**Affiliations:** 1 Fish Product Technology, Universitas Brawijaya, Malang, East java, 65145, Indonesia; 2 Bioseafood Research Unit, Universitas Brawijaya, Malang, East Java, 65145, Indonesia; 3 Department of Process and Food Engineering, Universiti Putra Malaysia, 43400 UPM, Serdang, Selangor, Malaysia; 4 Faculty of Sustainable Agriculture, Universiti Malaysia Sabah, Sandakan, 90509, Sabah, Malaysia

**Keywords:** Bruguiera gymnorrhiza, edible film, gelatin, physicochemical characteristics, silver nanoparticles

## Abstract

**Background:** Edible films intended for food packaging have been produced from hydrocolloids, lipids, resins, and composites, including gelatin. Gelatin is known to have a good filming ability and has been suggested as an alternative to non-biodegradable plastics. Naturally active compounds incorporated into film packaging may not only protect the food product from oxidation and microbial contamination, but they may also alter the physicochemical properties of the film. Silver nanoparticles have been used in food packaging as active agents due to their antibacterial and antifungal properties. The addition may affect the characteristics of the packaging. Therefore, this study aims to determine the effect of the addition of silver nanoparticles on the physicochemical characteristics of edible film from *Pangasius* sp. skin gelatin.

**Methods:** Mangrove extract of *Bruguiera gymnorrhiza* was used to synthesize silver into nanoparticle size. In this study, silver nanoparticles (AgNPs) with different concentrations (0, 2, 4 and 8%) were added into gelatin-based edible film. The edible films produced were observed for their physicochemical characteristics, including thickness, tensile strength, elongation, water vapour transmission, moisture content, pH, and colour.

**Results:** AgNPs affected the colour of the fish-gelatin-based edible film, as an increased concentration of AgNPs resulted in a darker film. Nevertheless, the addition of AgNPs showed no significant effect on the thickness (145–216 µm), tensile strength (14.58–19.72 MPa), elongation (21.86–54.19%), water vapour transmission (30.91–42.55 g/m ^2^/day), moisture content (9.57–11.16%) or pH (5.92–6.01) of the fish-gelatin-based edible film.

**Conclusions:** The addition of AgNPs has no significant effect on gelatin-based edible film physicochemical properties except colour. Therefore, the incorporated edible film has the potential to be developed further.

## Introduction

Due to their excellent structural properties and performances, many petrochemical-based plastics are widely used. However, since these materials are not deemed environmentally friendly, increased public awareness has led to the continued development of biodegradable packaging technology. ^1^ Edible film, one of the biodegradable packaging materials, is a thin layer attached to a food product and can be consumed. ^2^ Edible films can be made from various materials such as polysaccharides ( *e.g.*: starch, pectins, chitin and chitosan), lipids ( *e.g.*: bees wax, oils), and protein ( *e.g.*: whey, soy, pea protein, collagen, gelatin).

Gelatin is a protein macromolecule obtained from the hydrolysis of natural collagen found in skin, bone and animal connective tissue. ^3^ Usually, gelatin comes from cows or pigs, but using raw material sources for gelatin from these animals may cause some problems related to health and religious regulations. Alternatively, fish skin is considered a safer option as a raw material for gelatin production. ^4^ Pangasius catfish skin is a by-product resulting from the processing of fish fillets. The processing of *Pangasius* catfish fillets produces up to 55% of by-products, including fish skin. ^5^ The skin can potentially be used as a raw material for gelatin due to its abundant availability. Fish gelatin is now commonly used in the production of edible films due to its low melting temperature, low oxygen permeability, and good film-forming ability. ^6^

Nowadays, the concept of active packaging has attracted the food packaging industry and researchers. Active packaging may enable the interaction between the packaging, the food product, and the surrounding environment to improve the sensory properties and safety of food products. In addition, active agents with antimicrobial and antioxidant properties can help in extending the food’s shelf life and prevent food oxidation. ^7^ The edible film made using fish skin gelatin usually has low biological properties, so enriching it with active compounds is necessary to produce active packaging. Several studies have shown that edible films made from fish gelatin can be manufactured by adding several active ingredients. ^8^ One group of active ingredients that have the potential to be added are silver nanoparticles. Silver nanoparticles (AgNPs) are materials with at least one dimension with a size between 1 and 100 nm. ^9^ They can be made by synthesizing silver metal into nano-sized particles. This synthesis can be carried out with a biosynthesis biological agent. ^9^ In previous studies, the synthesis of silver nanoparticles from mangrove leaves such as *Excoecaria agallocha* L. ^10^ and *Rhizophora mucronata* ^11^^,^^12^ has been achieved. In this study, the biosynthesis process was done using the extract of mangrove leaves, *Bruguiera gymnorrhiza.* The content of bioactive compounds in plant extracts can be used as a bio-reductant that can convert silver metal into AgNPs. ^9^

Nurdiani *et al.,* ^13^ stated that AgNPs can improve the characteristics and functionalities of edible films, such as increasing product resistance, improving barrier properties as packaging and antimicrobial compounds. Therefore, this research was conducted to determine the physicochemical characteristics of edible film from Pangasius catfish skin gelatin with the addition of AgNPs of *Bruguiera gymnorrhiza.*

## Methods

### Preparation of gelatin

*Pangasius* sp. skin (frozen) was provided by PT. RUM Seafood, Sidoarjo Indonesia, and delivered to the Faculty of Fisheries and Marine Science, Universitas Brawijaya. Once the skins were thawed, they were cut into small pieces (0.5 × 0.5 cm ^2^) using clean scissors. The skins were stored in a freezer (−20°C) until use. Gelatin manufacturing started by soaking the *Pangasius* sp. skin in NaOH 0.1 M solution with a ratio of 1:5 (w/v) for two hours at room temperature. Afterwards, the skin was washed with running water until the pH was normal (pH = 7). Next, the skin was then soaked in an acetic acid solution with a concentration of 0.6 M at a ratio of 1:5 (w/v) for two hours (at room temperature). The swollen skin was then extracted with Aqua Dest, with a ratio of 1:3 (w/v) in a water bath (at 55–60°C) for four hours. Afterwards, the filtrate was collected and poured onto a baking tray for drying using a dehydrator for 8 to 12 hours at 55–60°C. The gelatin sheets obtained were then milled using a grinder to make gelatin powder. ^8^

### Preparation of mangrove extract

The leaves of *Bruguiera gymnorrhiza* were obtained from the Sendang Biru coastal area, Malang, East Java, Indonesia. The leaves were dried using a dehydrator for 8–12 hours at a temperature of 40–45°C. Dried leaves were then reduced in size using a grinder. Two grams of mangrove powder was then dissolved into 100 mL of deionized water and heated to boiling for three minutes. The mangrove solution was allowed to cool to room temperature, filtered using a Buchner funnel, and poured into a dark glass bottle. ^10^

### Preparation of silver nanoparticles (AgNPs)

One mL of mangrove leaf extract was mixed with 9 mL 20 mM AgNO _3_. The solutions were incubated for 0, 5, 10, and 15 minutes. The colour change was observed from the original whitish brown to brown. ^9^

### Production of edible films

Edible film was made by dissolving 4 grams gelatin into 100 mL distilled water. The gelatin solution was then heated at 50°C for 30 minutes. Next, the glycerol was added at 0.5% (v/v) and heated at 45°C for 15 minutes. AgNPs with the best incubation time was added at several concentrations per 100 mL (0 mL, 2 mL, 4 mL, and 8 mL). Once ready, the edible film solution was evenly poured into an 18 cm × 18 cm non-stick baking pan before being dried in an oven at a temperature of 55°C for 18–20 hours. The edible film was then removed from the oven, left at room temperature for 10 minutes, and peeled off slowly. ^13^

### Physicochemical characteristics analyses

*Thickness*

The thickness of the edible film sample was measured using a micrometre screw with an accuracy of 0.01 mm. Thickness measurements were carried out at five different points, and the average results represented the sample. ^14^

*Moisture content*

An empty crucible cup was oven dried at 105°C for 30 minutes. As it cooled down, 1 gram of the sample was then put into the cup and weighed (W1). Next, the sample was put into the oven and dried at a temperature of 105°C for three hours. After that, the cup was weighed again (W2). ^15^

$$\text{Moisture content}=\frac{W1-W2}{\text{Sample weight}}\times \;100\%$$

*Water vapour transmission rate (WVTR)*

The WVTR of each film was measured according to Ref. 16. The edible film was cut into a circle with a diameter of 3 cm. Ten grams of silica gel were poured into a cup. The sample was glued on top of the cup to cover it. The cup was weighed and recorded as W1. The sample was allowed to stand for 24 hours at room temperature and was then weighed again and recorded as W2. The WVTR was then calculated using the formula:

$$\text{WVTR}=\frac{\text{Weight difference}}{\text{Sample surface area}}\times \;\text{Time}$$

*Tensile strength and elongation*

Edible film was cut to a size of 5 × 1 cm ^2^. The thickness and initial length of the sample were measured. Next, each end of the edible film was clamped on a tensile machine. The sample was then pulled at 10 mm/min speed until it broke. ^14^ Finally, the tensile strength value was calculated by dividing the maximum stress (F max) by the area of the edible film using the formula:

$$\text{Tensile strength}=\frac{F\;\max}{A}$$

The measurement of the elongation of the edible film was carried out using a tensile strength device. Elongation is expressed as a percentage and is measured using the formula:

$$\text{Elongation}\;\left(\%\right)=\frac{\text{Stretch when breaking}\;\mathrm{up}\;\left(\mathrm{cm}\right)}{\text{Initial length}\;\left(\mathrm{cm}\right)}\times \;100\%$$

*pH*

The pH test was carried out using a pH meter. The measurement was conducted on the edible film solution prior to the drying process. ^17^

*Colour*

The colour of the edible films was measured using a colorimeter. Measurements were made by placing an edible film sample on the colour reader sensor. The reading button was set to L* (lightness), a* (redness) and b* (yellowness), and the target button was pressed. The results then appeared on the screen.

### Statistical analysis

SPSS commercial software (IBM SPSS Statistics version 25.0, Chicago, IL, USA) was used for statistical analysis. The data were expressed as mean ± standard deviation (mean ± SD). A one-way analysis of variance (ANOVA) and Tukey’s test with a p < 0.05 significance level were applied.

## Results and discussion

### Thickness of enriched edible film

Thickness is one of the characteristics of edible coatings that greatly affects the biological properties and shelf life of food-coating products. ^18^ This study showed that the addition of AgNPs into the gelatin solution had no significant effect (p > 0.05) on the thickness of edible film. The average thickness of the edible film was between 145 and 216 μm ( Table 1). According to the Japanese Industrial Standard (JIS), ^19^ the maximum thickness of edible film should be 250 μm. The thickness of the edible film is affected by the density, viscosity and surface tension of edible film materials as well as the drying time and layering technique. ^20^

**Table 1.** Physicochemical characteristics *Pangasius* sp. skin-gelatin-based edible film incorporated with AgNPs.

No	*Conc. of* AgNPs	Thickness (μm)	Tensile strength (MPa)	Elongation (%)	Water vapour transmission (g/m ^2^/day)	Moisture content (%)	pH
1	0%	145.50 ± 52.57 ^a^	19.72 ± 11.52 ^a^	21.86 ± 21.72 ^a^	37.09 ± 25.65 ^a^	9.57 ± 1.13 ^a^	6.01 ± 0.16 ^a^
2	2%	149.00 ± 57.51 ^a^	18.36 ± 5.14 ^a^	52.47 ± 41.28 ^a^	30.91 ± 14.46 ^a^	11.16 ± 1.97 ^a^	5.96 ± 0.11 ^a^
3	4%	163.00 ± 33.80 ^a^	15.67 ± 6.58 ^a^	54.19 ± 59.02 ^a^	42.55 ± 18.96 ^a^	10.40 ± 0.72 ^a^	5.95 ± 0.14 ^a^
4	8%	216.00 ± 21.78 ^a^	14.58 ± 5.88 ^a^	36.54 ± 38.35 ^a^	35.32 ± 29.79 ^a^	9.75 ± 1.81 ^a^	5.92 ± 0.15 ^a^

Results represent means ± standard deviation.

Values with the same superscript (a) within the same physochemical characteristic indicate no significant difference (p > 0.05).

Similarly, Arfat *et al*. ^19^ reported an increasing of edible film thickness due to the addition of silver–copper nanoparticles (Ag–CuNPs). The resulting range was 61–98 μm. The increase in thickness of the edible film was related to the increase of the solid content of the edible film. Furthermore, the addition of Ag–CuNPs to the gelatin affected the structure of the protein, causing it to become regular and the film network to be stronger.

### Moisture content of enriched edible film

The moisture content test was crucial to determine the total number of water molecules in the edible film tissue. The moisture content in the edible film affects the stability of the product, so it is expected that the moisture content in the edible film produced is low. This study showed that adding AgNPs into the gelatin solution had no significant effect (p > 0.05) on the moisture content of each edible film. The moisture content of the edible film had an average value of between 9.57 and 11.16% ( Table 1). Our result was lower than the study reported by Kanmani & Rhim. ^21^

Adding AgNPs into an edible film will usually increase its moisture content because of reduced interactions between gelatin chains and increased availability of free hydroxyl groups to absorb water. The moisture content of edible films can also be affected by temperature and drying methods. The temperature will affect the time needed in the drying process, causing an increase in the intensity of the interaction between water molecules and the hydrophilic polymer chain. ^22^

### WVTR

Statistically, the WVTR test indicated that adding AgNPs into the gelatin solution had no significant effect (p > 0.05) on the value of the WVTR of the edible film. The average value of the WVTR of the edible film produced was 30.91–42.55 g/m ^2^/day ( Table 1). Our results did not meet the Japanese Industrial Standard (JIS), ^23^ a maximum WVTR of 7 g/m ^2^/day. The high value of the WVTR of the edible films is due to the fact that the materials used to make the edibles are made of protein-derived materials that have polar polymers and a large number of hydrogen bonds, thus making the edible films capable of absorbing water at high humidity. The factors that can affect the WVTR values are the presence of chemicals and the structure of the constituent materials, the concentration of plasticizers, and environmental conditions such as humidity and temperature. ^24^

### Tensile strength and elongation

Table 1 shows that adding AgNPs into the gelatin-based edible film solution had no significant effect (p > 0.05) on the tensile strength of the edible film. The average tensile strength of the edible film was 14.58–19.72 MPa ( Table 1). The results are in accordance with the Japanese Industrial Standard (JIS), ^23^ as the minimum tensile strength of edible film should be 0.3 MPa. The value of the tensile strength of the edible films decreased with increasing concentrations of AgNPs. This could be because AgNPs are very weak in forming crosslinks with fish skin gelatin during the production of the edible films. In comparison, the elongation test results showed that adding AgNPs into the gelatin solution had no significant effect (p > 0.05) on the elongation of each edible film. The resulting edible film elongation value ranged from 54.19 to 21.86% ( Table 1). The results, however, were still much lower than the standard minimum elongation value of 70. ^23^ Elongation is inversely proportional to tensile strength. ^18^ According to Skurtys *et al.,* ^25^ elongation is expressed as a percentage change in the film’s original length before breaking and gives a measure of film elongation. Elongation values range from 1 to 80%. The level of elasticity of the edible coating can be seen from the elongation value; the higher the elongation value, the more elastic the edible coating.

The addition of AgNPs into the film resulted in a decrease in the tensile strength value. This might be due to the reduction in protein chain reactions due to incorporation with AgNPs. ^21^ The tensile strength of a film can be influenced by the constituent materials that have structural cohesion properties and the constituent materials of edible films, namely hydrocolloid protein (gelatin), contain amino acids capable of forming disulphide bonds. ^26^ In addition, the properties of the constituent materials to create a more robust gel network will help the formation of polysaccharide molecular structures more closely, forming a more coherent film structure and reducing the absorption of water molecules. ^27^ Bonilla & Sobral ^28^ stated that the elongation value of gelatin and chitosan edible films with ethanolic extracts from plants increased with the increased volume of extracts. This is due to the interaction between the phenolic compounds present in the extract. Furthermore, gelatin peptides can form covalent crosslinks, forming a more cohesive and flexible matrix. The higher the elongation value of the film, the more elastic it is so that the film can be stretched more. Meanwhile, films with low elongation values will be brittle.

### pH

The results showed that the pH of the enriched edible film ranged from 5.92 to 6.01 ( Table 1). It was indicated that the higher the concentration of AgNPs *Bruguiera gymnorrhiza* added, the lower the pH level of fish-gelatin-based edible film. *Bruguiera gymnorrhiza* mangrove leaf extract was detected to have an acidic pH of around 6.15, thus, the resulting edible film was expected to have a lower pH value than the control (0% AgNPs).

### Colour

Colour testing using a colour reader brings up three values: L*, a*, and b*. The L* value indicates the brightness level, a* indicates a red–green colour (a = +60 red, a = -60 green) and b* indicates a yellow–blue colour (b = +60 yellow, b = -60 blue). Edible films produced using gelatin usually have clear or dull/opaque colours. ^13^ Statistically, the addition of the concentration of AgNPs had a significant effect (p < 0.05) on the L*, a* and b* values ( Table 2).

**Table 2.** Colour value of edible film from *Pangasius* sp. skin gelatin enriched with AgNPs.

No	*Concentration of AgNPs*	L*	a*	b*
1	0%	87.363 ± 1.19 ^c^	0.440 ± 0.33 ^a^	1.978 ± 1.89 ^a^
2	2%	82.655 ± 1.39 ^bc^	2.223 ± 0.43 ^a^	7.508 ± 1.58 ^a^
3	4%	74.028 ± 5.41 ^ab^	5.833 ± 1.57 ^b^	13.890 ± 3.19 ^b^
4	8%	70.773 ± 6.36 ^a^	7.398 ± 2.63 ^b^	17.178 ± 3.99 ^b^

Results represent means ± standard deviation.

Values with different superscripts (a, ab, b, bc, c) indicate significant difference (p < 0.05) within the same colour value categories (L*, a*, b*).

The addition of a higher concentration of AgNPs on the fish-gelatin-based edible film resulted in a darker colour ( Figure 1), increasing the red and yellowish colour. Similarly, Shankar *et al.* ^29^ reported that the L* value of edible gelatin films with the addition of metallic nanoparticles (38.20) was lower than the control (93.15).

**Figure 1.**
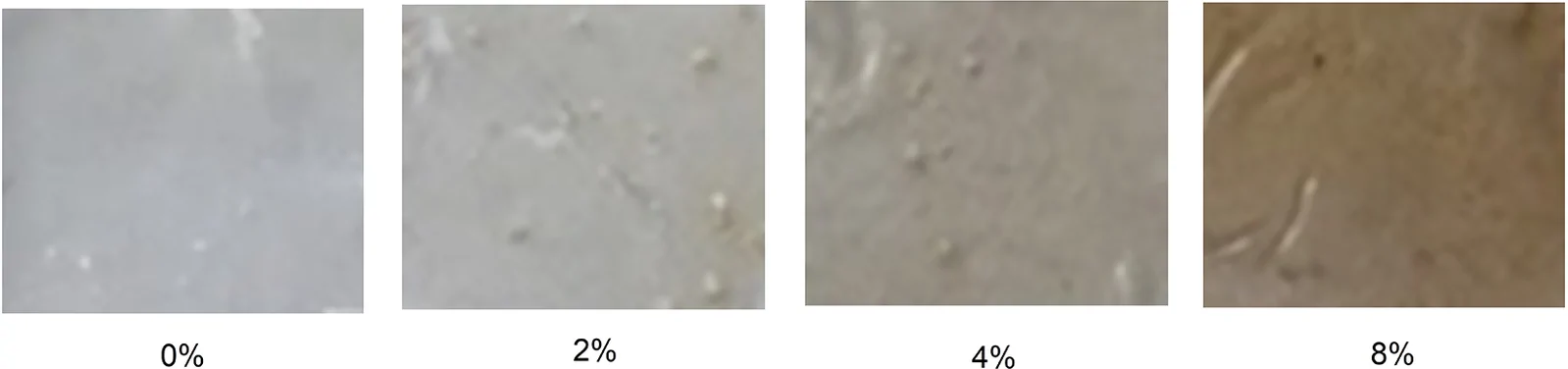
Appearance of enriched fish-gelatin-based edible film.

### The best treatment

The best treatment of edible film made from Pangasius skin gelatin with the addition of silver nanoparticles synthesized using *Bruguiera gymnorrhiza* extract was determined using the De Garmo method (grouping and weighting parameters, where the weights given are in accordance with the importance/priority of each parameter in affecting the results of the research). ^30^ Based on the calculation, it was suggested that the best treatment for all parameters was the third treatment (the addition of 4% silver nanoparticles ) ( Table 3).

**Table 3.** Physicochemical characteristics for the best treatment.

No	Parameter	*Edible film*	References
1	Thickness (μm)	163.0 ± 33.80	Max 250 ^23^
2	Tensile strength (MPa)	15.67 ± 6.58	Min 0.3 ^23^
3	Elongation (%)	54.19 ± 59.02	Min 70 ^23^
4	WVTR (g/m ^2^/day)	42.55 ± 18.96	Max 5 ^23^
5	Moisture content (%)	10.40 ± 0.72	Max 13 ^23^
6	pH	5.95 ± 0.14	5.75–6.60 ^31^
7	L* value	74.028 ± 5.41	38.20 ^29^
8	a* value	5.83 ± 1.57	15.08 ^29^
9	b* value	13.89 ± 3.19	12.24 ^29^

## Conclusion

The addition of various concentrations of AgNPs (0, 2, 4, and 8%) synthesized using mangrove *Bruguiera gymnorrhiza* had a significant effect on the colour of Pangasius gelatin-based edible film (L* value 70.773–87.363, a* value 0.440–7.398 and b* value 1.978–17.178). Nevertheless, adding AgNPs did not significantly affect the other physicochemical parameters (thickness, tensile strength, elongation, water vapour transmission, moisture content, and pH). It was suggested to use 4% AgNPs to get the best characteristics of the edible film based on the available standard. Based on the results, it is recommended to use AgNPs as active agents to produce active packaging since they have no unfavourable effect on the physicochemical properties of edible film. Further studies on the biological properties of edible film enriched with AgNPs is necessary.

## Ethical approval

This study did not require ethical approval because the authors used fish skins (available as waste or by-products) from the fish processing industry. Mangrove leaves were collected from a local mangrove conservation area in Malang, East Java. The conservation officer carried out the identification and collection of the mangrove leaves. The process was conducted in a way to ensures the sustainability of the local mangrove forest.

## Data Availability

Figshare: Physicochemical Characteristics of Pangasius sp. Skin Gelatin-based Edible Film Enriched with Silver Nanoparticles. https://doi.org/10.6084/m9.figshare.21640196.v2. ^32^ The project contains the following underlying data:
− DATA F1000 R NURDIANI.xlsx (raw data for colour, thickness, moisture content, water vapour transmission rate, pH, and tensile strength and elongation). Data are available under the terms of the Creative Commons Attribution 4.0 International license (CC-BY 4.0).
