# Supplementary material for: Physicochemical Characteristics of Pangasius sp. Skin-Gelatin-Based-Edible Film Enriched with Silver Nanoparticles
Source: F1000Res. 2026 Oct 5;12:160. Originally published 2023 Feb 10. [Version 3] doi: 10.12688/f1000research.129024.3 (PMC13684019; doi:10.12688/f1000research.129024.3)
Supplement: Peer review history

# Reviewer report: Reviewer response for version 3

**DOI:** 10.5256/f1000research.180246.r528098

**Published:** 2026-10-20

**Authors:** Andriati Ningrum

**Affiliations:** 1 Universitas Gadjah Mada, Yogyakarta, Indonesia

An evaluation and review of the manuscript, structured by section, based on the provided material:

**1. Manuscript Title & Overview**

- **Title:** *Physicochemical Characteristics of Pangasius sp. Skin-Gelatin-Based-Edible Film Enriched with Silver Nanoparticles*.
- **Authors:** Rahmi Nurdiani, Asep A. Prihanto, Muhamad Firdaus, Faridatul Nur Aini, Fajrin Adin Nabilah, Rosnita A. Talib, and Nurul Huda.
- **Core Focus:** Assessing the physical, mechanical, barrier, chemical, and optical properties of edible films formulated from Pangasius catfish skin gelatin incorporated with green-synthesized silver nanoparticles (AgNPs) using *Bruguiera gymnorrhiza* mangrove leaf extract.

**2. Abstract**

- **Summary:** The abstract outlines the global context of edible films as plastic alternatives, the green synthesis of AgNPs using mangrove leaf extract, the testing of four AgNP concentrations (0%, 2%, 4%, and 8%), and the evaluation of key physical/chemical parameters. It reports that AgNP addition significantly alters film color (darkening) but does not statistically alter thickness, tensile strength, elongation at break, water vapor transmission rate (WVTR), moisture content, or pH.
- **Critique & Evaluation:**
  - **Strengths:** Concise and well-structured, clearly stating the background, objective, methodology, evaluated parameters, and major quantitative ranges.
  - **Areas for Improvement:** The abstract frames AgNPs as active agents for antimicrobial food protection, yet the study evaluates only physicochemical characteristics. Explicitly noting in the abstract that biological efficacy and toxicity testing were outside the scope of this baseline study would clarify expectations for readers.

**3. Introduction**

- **Summary:** Provides a strong rationale by connecting environmental issues surrounding synthetic plastics with the valorization of fish processing waste (Pangasius fillet processing yields up to 55% by-products, including skin). It establishes the concept of active packaging, details the bioactive profile of *Bruguiera gymnorrhiza* mangrove extract (Taxonomy ID: 39984; antioxidant and antibacterial activity), and explains how plant bio-reductants form AgNPs.
- **Critique & Evaluation:**
  - **Strengths:** Well-grounded research problem, high industrial relevance regarding fish skin utilization, and clear justification for using green synthesis via mangrove extracts.
  - **Areas for Improvement:** Peer reviewers noted that the introduction initially lacked detail regarding the choice of the 2–8% AgNP concentration range and why antimicrobial activity was omitted despite introducing AgNPs as active agents. The authors later clarified that this work serves as a foundational physicochemical baseline before biological testing.

**4. Materials and Methods**

- **Summary:** Details the extraction of gelatin from Pangasius skin (NaOH pretreatment, acetic acid swelling, water bath extraction at 55–60°C), green synthesis of AgNPs from *B. gymnorrhiza* leaves (boiled extract mixed with 20 mM \(\text{AgNO}_3\)), and film casting using glycerol plasticizer (0.5% v/v) with drying at 55°C for 18–20 hours. Standardized characterization procedures are referenced for thickness, moisture content, WVTR (ASTM E), tensile strength/elongation, pH, and \(L^ *a^*b^*\) color values, followed by ANOVA and Tukey's post-hoc statistical analysis (\(\alpha = 0.05\)).
- **Critique & Evaluation:**
  - **Strengths:** Highly reproducible protocol with specific ratios, temperatures, times, and established standard testing methods. Ethical considerations regarding sustainable mangrove leaf harvesting and waste skin utilization are clearly stated.
  - **Areas for Improvement:** Methodological depth could be improved by including structural and microscopic characterization tools—such as Scanning Electron Microscopy (SEM), Fourier-Transform Infrared Spectroscopy (FTIR), or UV-Vis/DLS particle size analysis—to directly verify AgNP formation, particle size distribution, and spatial dispersion within the gelatin matrix.

**5. Results and Discussion**

- **Summary:**
  - **Physicochemical Parameters:** AgNP incorporation (0–8%) resulted in non-significant statistical differences (\(p > 0.05\)) for thickness (145.50–216.00 \(\mu\text{m}\)), tensile strength (14.58–19.72 MPa), elongation at break (21.86–54.19%), WVTR (30.91–42.55 \(\text{g/m}^2/\text{day}\)), moisture content (9.57–11.16%), and pH (5.92–6.01).
  - **Color:** Showed significant changes (\(p < 0.05\)), with lightness (\(L^*\)) decreasing from 87.36 to 70.77, redness (\(a^*\)) increasing from 0.44 to 7.40, and yellowness (\(b^*\)) increasing from 1.98 to 17.18 as AgNP concentration increased.
  - **Compliance with Standards:** Tensile strength, thickness, and moisture content met Japanese Industrial Standards (JIS). However, elongation at break fell below the minimum JIS requirement (54.19% vs. \(\ge 70%\)) and WVTR exceeded the maximum threshold (\(30.91\text{--}42.55 \text{ g/m}^2/\text{day}\) vs. \(\le 7 \text{ g/m}^2/\text{day}\)) due to the hydrophilic nature of gelatin proteins.
  - **Optimal Formulation:** The De Garmo weighting method identified 4% AgNP addition as the best overall treatment.
- **Critique & Evaluation:**
  - **Strengths:** Transparent presentation of tabular data, honest comparison against industrial standards, and logical explanation of protein-water interactions explaining high WVTR values.
  - **Areas for Improvement:** As highlighted during peer review, despite reporting no statistical significance (\(p > 0.05\)), several parameters show large numerical variations (e.g., a 32% numerical increase in thickness at 8% AgNP and large standard deviations in elongation, such as \(54.19 \pm 59.02%\)). High standard deviations suggest sample variability or small sample size, which warrants re-evaluating statistical power or expanding sample replicates.

**6. Conclusions**

- **Summary:** Concludes that green-synthesized AgNPs significantly alter edible film color without negatively impairing major physicochemical properties. Recommends 4% AgNP incorporation as optimal while explicitly stating that future research is required to evaluate toxicity, biological activity (antimicrobial efficacy), and functional performance in food packaging applications.
- **Critique & Evaluation:**
  - **Strengths:** Directly aligns with the presented data and explicitly outlines the key limitations and necessary next steps regarding safety and biological testing.
  - **Areas for Improvement:** Practical claims regarding active food packaging suitability remain tentative until migration studies, toxicity evaluations, and real food shelf-life trials are performed.

Is the work clearly and accurately presented and does it cite the current literature?

Yes

If applicable, is the statistical analysis and its interpretation appropriate?

Yes

Are all the source data underlying the results available to ensure full reproducibility?

Yes

Is the study design appropriate and is the work technically sound?

Yes

Are the conclusions drawn adequately supported by the results?

Yes

Are sufficient details of methods and analysis provided to allow replication by others?

Yes

Reviewer Expertise:

food science

I confirm that I have read this submission and believe that I have an appropriate level of expertise to confirm that it is of an acceptable scientific standard.

# Reviewer report: Reviewer response for version 3

**DOI:** 10.5256/f1000research.180246.r527457

**Published:** 2026-10-12

**Authors:** Amiza Mat Amin

**Affiliations:** 1 Faculty of Fisheries and Food Science, Universiti Malaysia Terengganu, Kuala Nerus, Malaysia

I have no further comment to make.

Is the work clearly and accurately presented and does it cite the current literature?

Yes

If applicable, is the statistical analysis and its interpretation appropriate?

Yes

Are all the source data underlying the results available to ensure full reproducibility?

Yes

Is the study design appropriate and is the work technically sound?

Partly

Are the conclusions drawn adequately supported by the results?

Yes

Are sufficient details of methods and analysis provided to allow replication by others?

Yes

Reviewer Expertise:

food science, food protein

I confirm that I have read this submission and believe that I have an appropriate level of expertise to confirm that it is of an acceptable scientific standard.

# Reviewer report: Reviewer response for version 2

**DOI:** 10.5256/f1000research.168310.r319877

**Published:** 2024-09-20

**Authors:** Abhilash Sasidharan

**Affiliations:** 1 Kerala University of Fisheries and Ocean Studies, Kerala, India; 2 Norwegian University of Science and Technology (NTNU), Trondheim, Norway

The study is significant considering the topic selected. The replacement of synthetic plastics in food packaging is important in connection to the sustainable developmental goals targeted to be achieved in near future.

 This study focusses on the effect of silver nanoparticles on a fish gelatine based edible film. The study concludes that the AgNPs had no significant effect on any of the physicochemical properties.

 AgNPs are well known for their antimicrobial properties which is not discussed in this study.

 As the study proposes edible application, the toxicity of the film also needs to be discussed in details. Noting that the application percentage is as high as 8%, it needs to be either discussed in detail or demonstrated using toxicity evaluation.

 All the values are statistically reported as homogenous with no significant effect. The thickness of the films compared to the control film has increased 32% in 8% AgNP incorporated film. Similar differences in values are evident in many other parameters as well.

 Hence the statement “This study showed that the addition of AgNPs into the gelatin solution had no significant effect (p > 0.05) on the thickness of edible film” needs to be reevaluated.

 The article needs to be modified regarding the significant level statements and the statistical analysis has to be redone.

Is the work clearly and accurately presented and does it cite the current literature?

Yes

If applicable, is the statistical analysis and its interpretation appropriate?

No

Are all the source data underlying the results available to ensure full reproducibility?

No source data required

Is the study design appropriate and is the work technically sound?

Partly

Are the conclusions drawn adequately supported by the results?

Partly

Are sufficient details of methods and analysis provided to allow replication by others?

Yes

Reviewer Expertise:

NA

I confirm that I have read this submission and believe that I have an appropriate level of expertise to state that I do not consider it to be of an acceptable scientific standard, for reasons outlined above.

# Response

**Published:** 2024-11-15

**Authors:** Rahmi Nurdiani

**Affiliations:** Universitas Brawijaya, Indonesia

Responses to Reviewer 3

 Comment 1

 AgNPs are well known for their antimicrobial properties which is not discussed in this study.

 Response 1

 This study primarily focuses on examining the physicochemical properties of AgNPs as a foundational investigation. Given its preliminary nature, the research does not cover the antimicrobial properties, despite the known effectiveness of AgNPs in this area. Instead, this study aims to establish baseline data on physicochemical characteristics, paving the way for future studies to delve into antimicrobial effects and applications.

 Comment 2

 As the study proposes edible application, the toxicity of the film also needs to be discussed in details. Noting that the application percentage is as high as 8%, it needs to be either discussed in detail or demonstrated using toxicity evaluation.

 Response 2

 As this study is preliminary and focuses primarily on the physicochemical properties of the film, a toxicity test was not included in the scope of this research. The primary objective was to establish baseline data on the film’s properties, which is essential groundwork before moving into applications and safety assessments. Future research is necessary to investigate toxicity in detail, particularly for edible applications, and this study paves the way for such in-depth evaluations.

 Comment 3

 All the values are statistically reported as homogenous with no significant effect. The thickness of the films compared to the control film has increased 32% in 8% AgNP incorporated film. Similar differences in values are evident in many other parameters as well. Hence the statement “This study showed that the addition of AgNPs into the gelatin solution had no significant effect (p > 0.05) on the thickness of edible film” needs to be reevaluated.

 Response 3

 Even though the trends in thickness and other parameters appear different—such as the observed 32% increase in thickness in the 8% AgNP-incorporated film compared to the control—the statistical analysis indicated that the differences are not significant (p > 0.05). This suggests that, despite visible variations, the changes are likely due to natural variability and do not reflect a consistent, reliable effect of AgNP incorporation on film thickness.

# Response

**Published:** 2026-09-14

**Authors:** Rahmi Nurdiani

**Affiliations:** Universitas Brawijaya, Indonesia

**Comment 1**

 AgNPs are well known for their antimicrobial properties which is not discussed in this study.

**Response 1**

 This study primarily focuses on the physicochemical properties of the AgNP-enriched edible film as a foundational investigation. Given the preliminary nature of the study, antimicrobial activity was not evaluated in the present research, although AgNPs are well known for their antimicrobial properties. Instead, this study aims to establish baseline data on the physicochemical characteristics of the developed film, providing a basis for further investigations into its antimicrobial activity and potential applications. Accordingly, we have added this explanation to the last paragraph of the Introduction to clarify the scope and rationale of the present study.

**Comment 2**

 As the study proposes edible application, the toxicity of the film also needs to be discussed in details. Noting that the application percentage is as high as 8%, it needs to be either discussed in detail or demonstrated using toxicity evaluation.

**Response 2**

 As this study was preliminary and primarily focused on the physicochemical properties of the film, toxicity testing was beyond the scope of the present research. The main objective was to establish baseline data on the film properties, which provides essential groundwork for subsequent application and safety assessments. We agree that toxicity evaluation, particularly for potential edible applications, is an important aspect of future research. Accordingly, we have added a statement in the Conclusion section recommending further studies to comprehensively evaluate the toxicity and safety of the developed film.

**Comment 3**

 All the values are statistically reported as homogenous with no significant effect. The thickness of the films compared to the control film has increased 32% in 8% AgNP incorporated film. Similar differences in values are evident in many other parameters as well. Hence the statement “This study showed that the addition of AgNPs into the gelatin solution had no significant effect (p > 0.05) on the thickness of edible film” needs to be reevaluated.

**Response 3**

 Although the trends in thickness and other parameters appeared to differ among treatments, statistical analysis indicated that these differences were not statistically significant (p > 0.05). Thus, despite the observed numerical variations, the changes may reflect natural variability rather than a consistent and statistically supported effect of AgNP incorporation on film thickness. Accordingly, we have made several adjustments to the reporting and presentation of the results.

# Reviewer report: Reviewer response for version 2

**DOI:** 10.5256/f1000research.168310.r306972

**Published:** 2024-08-08

**Authors:** Amiza Mat Amin

**Affiliations:** 1 Faculty of Fisheries and Food Science, Universiti Malaysia Terengganu, Kuala Nerus, Malaysia

The authors have done the required correction.

Is the work clearly and accurately presented and does it cite the current literature?

Yes

If applicable, is the statistical analysis and its interpretation appropriate?

Yes

Are all the source data underlying the results available to ensure full reproducibility?

Yes

Is the study design appropriate and is the work technically sound?

Partly

Are the conclusions drawn adequately supported by the results?

Yes

Are sufficient details of methods and analysis provided to allow replication by others?

Yes

Reviewer Expertise:

food science, food protein

I confirm that I have read this submission and believe that I have an appropriate level of expertise to confirm that it is of an acceptable scientific standard.

# Reviewer report: Reviewer response for version 1

**DOI:** 10.5256/f1000research.141678.r166792

**Published:** 2024-05-28

**Authors:** Amiza Mat Amin

**Affiliations:** 1 Faculty of Fisheries and Food Science, Universiti Malaysia Terengganu, Kuala Nerus, Malaysia

The manuscript described the effect of incorporating silver nanoparticles in the silver catfish gelatin film. The introduction is sufficient. Please state why the range of 2-8% was used in this study. However, this study did not include antimicrobial activity of the gelatin films.  Please comment why there was no significant difference in most results.

Is the work clearly and accurately presented and does it cite the current literature?

Yes

If applicable, is the statistical analysis and its interpretation appropriate?

Yes

Are all the source data underlying the results available to ensure full reproducibility?

Yes

Is the study design appropriate and is the work technically sound?

Partly

Are the conclusions drawn adequately supported by the results?

Yes

Are sufficient details of methods and analysis provided to allow replication by others?

Yes

Reviewer Expertise:

food science, food protein

I confirm that I have read this submission and believe that I have an appropriate level of expertise to confirm that it is of an acceptable scientific standard, however I have significant reservations, as outlined above.

# Response

**Published:** 2024-07-16

**Authors:** Rahmi Nurdiani

**Affiliations:** Universitas Brawijaya, Indonesia

Responses to Reviewer 2

**Comment 1**: 

 Please state why the range of 2-8% was used in this study. However, this study did not include antimicrobial activity of the gelatine films.

**Response 1**:

 The concentration of silver nanoparticles (AgNPs) added to the edible film ranged from the minimum amount needed to show antibacterial activity to the maximum amount considered safe.

**Comment 2:**

 Please comment why there was no significant difference in most results.

**Response 2:**

 The physicochemical properties of edible films enriched with various concentrations of silver nanoparticles were evaluated. The addition of silver nanoparticles, regardless of the concentration, did not significantly affect any of the physicochemical parameters evaluated (Table 1). This might be because the concentration of silver nanoparticles added was very small. Similar to the study conducted by Gutiérrez et al. 24, which investigated the impact of adding calcium and silver nanoparticles to gelatin-based films. They found that the films' structural integrity and mechanical properties were preserved. The dispersion of AgNPs within the gelatin matrix was uniform, preventing significant changes in the film's tensile strength and elasticity. Additionally, the barrier properties, such as water vapour permeability, remained stable.

# Reviewer report: Reviewer response for version 1

**DOI:** 10.5256/f1000research.141678.r208387

**Published:** 2023-10-10

**Authors:** Mohamed Bilal Goudjil

**Affiliations:** 1 Applied Sciences Faculty, Process Engineering Laboratory, KASDI Merbah-Ouargla University, Ouargla, Algeria

This is interesting original research that aims to investigate the Physicochemical characteristics of *Pangasius* sp. skin-gelatin-based-edible film enriched with silver nanoparticles.

 Overall, the manuscript is well complied and experimental data is appreciable. However, a few comments below must be addressed by the authors with utmost attention before considering for indexing.

 1. In introduction section: Please give some information about the plant used in the present study.

 2. Author should provide herbarium number or plant code for the *Bruguiera gymnorrhiza* used.

 3. In the Results and discussion section, a brief talk about the reasons for the formation of nanoparticles using green synthesis, specifying the mechanism and the responsible component, is required.

 4. The conclusion is a bit poor, it should be improved to reflect the work done.

 5. I believe the language can be more polished by the authors.

Is the work clearly and accurately presented and does it cite the current literature?

Yes

If applicable, is the statistical analysis and its interpretation appropriate?

Partly

Are all the source data underlying the results available to ensure full reproducibility?

No source data required

Is the study design appropriate and is the work technically sound?

Partly

Are the conclusions drawn adequately supported by the results?

No

Are sufficient details of methods and analysis provided to allow replication by others?

Yes

Reviewer Expertise:

Phytochemistry, Essential oil, Chromatography, Renewable Energies, Nanotechnology

I confirm that I have read this submission and believe that I have an appropriate level of expertise to confirm that it is of an acceptable scientific standard, however I have significant reservations, as outlined above.

# Response

**Published:** 2024-07-16

**Authors:** Rahmi Nurdiani

**Affiliations:** Universitas Brawijaya, Indonesia

**Comments from the Reviewers**

**1** **^st^** **REVIEWER**

**Comment 1**: In introduction section: Please give some information about the plant used in the present study.

**Response 1**:

*Bruguiera gymnorhiza,* a member of the *Rhizophoraceae* family, is characterized by its glabrous and relatively smooth trunk with reddish-brown bark. This species is typically found along the seaward margins of mangrove swamps. *B. gymnorhiza* leaf extract has demonstrated potential as an active compound for incorporation into fish gelatin films, exhibiting inhibitory effects against various pathogenic bacteria such as *Pseudomonas aeruginosa* (5.77 - 23.13 mm), *Aeromonas hydrophila* (14.17 - 23.47 mm), and *Staphylococcus aureus* (1.70 - 4.43 mm) (Effendi et al., 2023). Additionally, the leaf extract has shown significant radical scavenging activity, with an IC _50_ value of 12.93 µg/mL (Indriaty et al., 2023).

**Comment 2:**  Author should provide herbarium number or plant code for the *Bruguiera gymnorrhiza *used.

**Response 2: **The leaves of *Bruguiera gymnorrhiza* (Taxonomy ID: 39984) were obtained from the Sendang Biru coastal area, Malang, East Java, Indonesia.

**Comment 3:** 

 In the Results and discussion section, a brief talk about the reasons for the formation of nanoparticles using green synthesis, specifying the mechanism and the responsible component, is required.

**Response 3:**

 The physicochemical properties of edible films enriched with various concentrations of silver nanoparticles were evaluated. In this study, silver nanoparticles were synthesized using green synthesis with mangrove leaf extract. The reason for the formation of nanoparticles using green synthesis for active agents incorporated in gelatin edible films is biocompatibility. Green-synthesized nanoparticles are often biocompatible and non-toxic, making them suitable for use in edible films that come into direct contact with food products.22 The green synthesis of nanoparticles using plant extracts involves a mechanism that includes the reduction of metal ions, complex formation, stabilization, and shape control. This process utilizes the natural compounds present in plant extracts to facilitate the synthesis of nanoparticles with unique properties.23

**Comment 4:** The conclusion is a bit poor; it should be improved to reflect the work done.

**Response 4:**

 The addition of various concentrations of AgNPs (0, 2, 4, and 8%) synthesized using mangrove *Bruguiera gymnorrhiza* had a significant effect on the colour of Pangasius gelatin-based edible film (L* value 70.773–87.363, a* value 0.440–7.398 and b* value 1.978–17.178). Nevertheless, adding AgNPs did not significantly affect the other physicochemical parameters (thickness, tensile strength, elongation, water vapour transmission, moisture content, and pH). This study recommends using 4% AgNPs to achieve the best qualities in edible film, meeting established standards. The results suggest that AgNPs can be effective active agents for developing active packaging without negatively affecting the film's physicochemical properties. However, further research is needed to explore the biological functions of edible films with added AgNPs.

**Comment 5:**

 I believe the language can be more polished by the authors.

**Response 5:**

 Regarding the grammatical and sentence construction errors, we have revised our manuscript.

# Response

**Published:** 2024-07-26

**Authors:** Mohamed Bilal Goudjil

**Affiliations:** process engineering, Kasdi Merbah Ouargla University, Ouargla, Ouargla Province, Algeria

The required corrections have been made, the work is now acceptable.
